# The Anti-vivisection Agitation

**Published:** 1898-12

**Authors:** E. V. Wilcox

**Affiliations:** Professor of Biology, Montana State College


### THE ANTI-VIVISECTION AGITATION.

BY E. V. WILCOX, PH.D.,
PROFESSOR OF BIOLOGY, MONTANA STATE COLLEGE.

(Concluded from page 720.)

WE come next to consider another main characteristic of anti-vivisection literature, namely, that of abusive language toward scientists.

In their use of language the anti-vivisectionists, as we might expect from their other methods of agitation, do not hesitate to stoop to the level of the ordinary street bully.

We find certain vivisectors called "inhuman devils," and these words are used as a title of an anti-vivisection circular. The circular contains a childish attempt at a justification of their use. A national petition blank, "for the total abolition of vivisection," gives the following definition:

"Vivisection is the cutting, poisoning, burning, freezing, smothering, and breaking the bones of live animals by medical scientists, and is done all over the world."

No honest or sincere person would be guilty of such an absurdly malicious misrepresentation of their opponents' position. One man says that vivisection "is useless, wicked, cruel, barbarous, and infamous," and this twaddle is repeated *ad nauseam*. Vivisectors are said to be in league with the devil, their knowledge is said to be "diabolical" and "diabolism." Anti-vivisectionists tell us that our schools are turning out "young devils." Our schools are called "hells of cruelty," and several other kinds of "hells." If our schools are "hells," it would follow as a corrollary that

"young devils" would be turned out. Vivisectors are called "fiends," "hell-fiends," their work is said to be "fiendish." Vivisection, they say, is an "atrocious crime" for which there is no penalty too great, "certainly none that is not deserved."

We hardly need to remind ourselves that when the anti-vivisectionists went before the Massachusetts Legislature and Congress they were utterly unable to present a single fact to substantiate the charges made in their circulars. They could not instance a case of the abuse of vivisection. The whole fabric of anti-vivisection literature is simply the product of morbid imagination—simply "words, words, words."

We have now to consider another feature of anti-vivisection literature, namely, its sensationalism and its consequent demoralizing influence.

This is by far the most serious defect of the literature of anti-vivisectionists, a defect in comparison with which their ignorance, false statements, and abusive language sink into insignificance. Their ignorance only makes them the laughing-stock, as they are, of sensible people. Their false statements merely win over to their side a few uninformed persons. Their abusive language harms no one but themselves. But the greater number of their circulars and tracts, so highly sensational that no reputable periodical would publish them, and therefore published by themselves, threaten to become a veritable curse to the nation. These sensational accounts are all greatly exaggerated, and most of them are delusions of the imagination or confessedly taken from the testimony of janitors and from "horrid rumors concerning the practice of vivisection in the schools." Many pretended "revelations" are devoid of sense, reason, and sanity.

Furthermore, there is no demand for this sort of literature except in so far as the anti-vivisectionists have themselves created it. They have substantiated none of their charges, have never shown any reason why they should undertake the present crusade. Notwithstanding all this, anti-vivisectionists tell us that there are one hundred and two societies in this country and in Europe which are organized for the express purpose of circulating sensational literature on the subject of vivisection. This, we submit, is a blot upon the civilization of our age.

Is it not enough that the newspapers of the land give us the sickening details of every murder, suicide, and accident; that countless thousands of detective stories and the like are circulated all over the country? Are we in need of more such stuff? Nor should we forget the ape-like imitative nature of man. No suicide is committed but it has a goodly crop of imitators; no murder done but some one duplicates it. Newspaper men do not justify themselves on moral grounds, much less do they set up the childish claim of the anti-vivisectionists that they are carrying on a moral crusade against vice. Newspaper editors tell us that they have to write sensational material in order to compete with other papers. It is a question of dollars and cents with them. The anti-vivisectionists, however, do not have even that excuse. They have deliberately founded societies and despatched their sensation-hunters to various parts of this country and even to Europe. They have solicited money from various unsophisticated persons as a means of publishing their brainsickly accounts.

The anti-vivisectionists have left no stone unturned in their search after startling sensations. They not only prowl about various laboratories with morbid curiosity, but they read the publications of physiological laboratories with the hope of finding something sufficiently exciting for one of their circulars. They are unable to appreciate the work of a physiologist, and cannot understand physiological literature. They simply select those sentences which, to a diseased imagination, savor of the sensational, deliberately omitting the setting of the sentences quoted, and then publish these statements with innumerable additions, exaggerations, and material of their own manufacture. This is the sort of literature with which the anti-vivisectionists are corrupting the youth of the country.

Scientific publications are written for scientists, not for imbeciles, children, and quack-doctors, nor even for the general public.

The people for whom these publications are intended bring to their perusal an understanding of the subject and an honest purpose of reading them for the scientific information which they contain. These publications are of extreme usefulness; are, indeed, necessary to the scientific worker. Physicians, also, must know and have publications on various medical matters which no sane man would think of publishing broadcast in popular tracts.

Publications on experimental physiology are of a very technical nature, and are unintelligible even to the ordinary scientist, except he be a specialist in this field. Anti-vivisectionists tell us that all that is necessary to appreciate this literature is a slight acquaintance with physiological terms. Impenetrable ignorance cannot appreciate its own darkness.

In the sensational accounts of the anti-vivisectionists we have abundant evidence of mental pathology, interesting material for the specialist in nerve-diseases. Their pages are filled with various suggestions as to the criminal possibilities of vivisection. Such ideas never entered the brain of a vivisector or any sound brain, and would never have been thought of had it not been for certain brainsick anti-vivisectionists. These delusions of mental obliquity (falsehoods, they would be called in the case of responsible persons) are printed privately and circulated throughout the country in countless numbers. Various absurd cruelties are imputed to vivisectors. Anti-vivisectionists are putting this morbid literature into the hands of the youth. The effect is already shown in various criminal practices among boys who have simply carried out the suggestions contained in the anti-vivisectionist circulars. The anti-vivisectionists, however, are not content with merely suggesting to our youth various crimes which would otherwise not have occurred to anyone, but whenever a boy has acted upon their suggestion they have in each case ferreted out the whole affair and published it in their circulars with all the nauseating details and with all the other characteristically anti-vivisectionist embellishments. Such are the experiments which the anti-vivisectionists are trying on the youth of our country. Such are what they are pleased to call their "experiments on human patients." And yet, to quote from one of their tracts, we see these people "mingling socially with their fellows, but described in Holy Writ as 'whited sepulchres, which, indeed, appear beautiful outwardly, but within are full of dead men's bones and all uncleanliness.'" To such insane lengths does this unholy passion for sensations extend that men "who already have a reputation for this diabolical sport" have actually gone abroad to work up scandals for importation, and "with an effrontery that would shame the veriest mounteback they still delude the credulous public with the same old falsehoods." "It will not do to tamper with this vice" of corrupting the young. "Restriction is not enough. We do not attempt to mildly restrict thieves and highway assassins. The only hope is to stamp the whole practice as what it really is—an atrocious crime —and then make and enforce laws that will punish it. There can hardly be too strong a law, or too great severity of penalty, certainly none that is not deserved." I thank you, anti-vivisectionists, for teaching me those words.

A good illustration of the imbecility of anti-vivisectionists is given in their idea of a course in medicine. Vivisection would, of course, have no place in their school. Physiology, the most important branch of medical work, could therefore be taught merely from books. We could show, also, by numerous quotations from their tracts that they are opposed to dissection. Moreover, the only medical school of which they seem to approve advertises in the following manner: "This school advocates a new and successful method of acquiring a medical education, and boldly proclaims itself the champion of the struggling yet would-be physician, and announces that any student who has attained a sufficient mastery of the art of healing to pass the searching examination of our institution is entitled to receive its diploma, even though all his knowledge has been attained by practice and by burning the midnight oil in the solitude of his own home. We have established a system of study which bears the same relation to medicine as the Chautauqua Association does to literature, by which the diligent (sic) student may intelligently progress as rapidly as his talent will allow without reference to any time-limit. Successfully establishing this idea has opened the way to a profession to many a brilliant mind whose environments were such that they would otherwise have been debarred from working in the field of medical science for the benefit of their fellow-men. Students can enter at any time; can receive instruction daytime or evening the year around. Private lectures given to those desiring to advance more rapidly toward graduation. Lectures (sic) and books can be sent to students taking the course by mail. Prompt and special attention given to this department of our work."

So far as we are informed, the department for removing corns and performing surgical operations by mail has not yet been established, but we do not doubt that out-patients can receive treatment by mail. In order to make clearer the meaning of the abstruse advertisement, we must translate it. The translation reads as follows: "This school advocates a new and patent method of acquiring certain medical formulæ; charlatan-like, proclaims itself the champion of the embryo quack, and announces that any person who has sufficiently mastered the black arts to pass our examination in those arts is entitled to receive our diploma, although he may be scarcely qualified for a butcher's apprentice, and although he may have acquired his modicum of knowledge by merely practising on poor unfortunate victims, experimenting on human patients, and by smoking cigarettes in the solitude of his own room. We have established a system of study which bears the same relation to medicine as faking does to legitimate business, by which the *diligent* person may progress in the science of quackery as fast as his peculiar aptitude to this business permits. Successfully establishing this idea has opened the way to a fortune for many a clever fellow, who otherwise would have remained an ordinary fakir, and who would have been debarred from entering the field of medical charlantism for the destruction of his fellowmen. Instruction at all hours day or night the year around. Special hints given to those desiring to do advanced work in medical trickery. *Lectures* and books sent in registered packages to those taking the course by mail."

But it is impossible to make a parody of such an advertisement. It is itself a parody. Nothing could be more bombastic and ludicrous. It is simply the method which is universally adopted by charlantism. Moreover, by studying the literature of the anti-vivisectionists we find other interesting features in their idea of a medical course. We read: "For malaria, bacteria, contagion, etc., should be substituted mental causation, such as grief, fear, doubt, jealousy, envy, and greed." "Metaphysics should constitute the leading feature in courses of medical study." "Devils" is not the word for graduates from such a school. Bombast and ignorance, mixed in about equal proportions, as they would be in their graduates, produce charlatans. Yet this is for the anti-vivisectionists the standard of medical science. According to their idea, as set forth in their own tracts, a model physician would be one who never vivisected, never dissected; simply took a course by mail, and that with metaphysics as its main feature. With regard to such a medical school we have but one remark to add: We hope we may never be among the victims of their work in "experiments on human patients."

The anti-vivisectionists have sent out a circular[1] purporting to reproduce written "reports" from their supporters in various States. We here reproduce a few of them.

"We are told that thirty cats have been 'cut up' in a certain school in Wisconsin the past winter. Do the parents of the pupils know it, and not interfere? If so, they cannot be surprised when 'cut up' themselves in their old age. Of course not!"

"One girl told me that in her class at high school the teacher, a man (?) wanted to show the effect of atmospheric pressure in and on all things, and that he put a mouse in an air-receiver, exhausted all the air until the mouse swelled up with all the awful pressure inside of him, jumped up and down in vain attempts to grasp at

[1] "Biology" in the public schools. Illinois Anti-vivisection Society.

the receding air, and finally burst *all to pieces.* Is not this too horrible to believe?"

Evidently nothing is "too horrible" for an anti-vivisectionist to believe, and the more impossible the piece of gossip is the more firmly he believes it. The above-quoted bit of nonsense is, as anyone who ever saw such an experiment knows, absolutely impossible. But we have to remember that to the anti-vivisectionist the impossible is the absolute truth.

Complaints are made to me that in various Massachusetts cities and towns boys and girls are being taught in the public schools to *dissect male and female cats.* The italics are in the original. This statement is termed by the anti-vivisectionists a "revelation." The writer well remembers the time when this solemn announcement first appeared in the Boston papers, and what a roar of laughter it created among the students. Are the cats to be blamed for being "male and female?" Or does the witness, at this late hour, wish to reprove the Creator for creating animals male and female? Perhaps we are dealing with another occultist who could furnish astral cats without sexuality! The immorality of the whole affair does not lie in the fact of the cats being male and female, but in the suggestive indecency of the witness's remark. We have here simply a case of sexual psychopathy. It would never occur to a normal person to call attention to the sexuality of animals in such a lascivious manner. A man who cannot speak of the reproductive functions of animals without insinuating the indecency of those functions is most certainly to be pitied.

The contention of the anti-vivisectionists, that experimentation on animals has never resulted in the discovery of truth, is simply a denial of all that is known of physiology and infectious diseases, and is unworthy of notice.

After an unceasing agitation for over a quarter of a century, the anti-vivisectionists inform us that the "leading physicians" condemn vivisection. Several quotations from the statements of these "leading physicians" have been made above. The quotations give us an idea of the character of these "leading" minds. With all their urging, inducements, and exaggerated tales, the anti-vivisectionists have been able to obtain anti-scientific testimonials from five out of every thousand physicians in this country! This is interesting as showing the proportion of physicians who are as yet unacquainted with the work in experimental physiology. A few medical schools with correspondence courses, and with metaphysics as the chief study, should be able to swell the ranks of those "leading physicians" who know nothing of experimental science. In order to become a "leading physician" one has merely to say that vivisection is "useless, wicked, cruel, barbarous, and infamous," whereupon he at once acquires the title "leading physician," and has the great pleasure of seeing a photograph of himself in the anti-vivisectionists' journals. No one could hit upon a cheaper advertising device or one more nearly on a level with the testimonial-and-advertisement scheme of patent-medicine concerns. In the world of genuine science and medicine a man can acquire fame only by honest and successful labor. In the circulars of the anti-vivisectionists that man is chief who has shown himself to be the most expert in the use of abusive epithets and the most successful in the anti-vivisectionists' favorite amusement of hunting after sensational matter.

Their latest agitation is an attempt to pass a law which will prevent the Bureau of Animal Industry from experimenting on the infectious diseases of animals. As usual, they have tried to hoodwink the public with a careful wording which seems to imply only a "restriction" of experimental work. But, as clearly shown by Dr. Charles W. Dabney, Jr., and Secretary Wilson, the effect of the bill, if passed, would be the complete prohibition of the most useful of the experiments of that bureau. Moreover, the anti-vivisectionists themselves admit that they wish this proposed legislation to be an "entering wedge," and from their own circulars we see that they are opposed to dissection as well as vivisection, and in every form. The time has come when the commonsense of the country should administer the proper rebuke to these violent agitators.

And now we must close the anti-vivisectionists' side of the case with the finding that they have "no case." Their monstrous heap of sensational rubbish is a mere mass of the products of ignorance, prejudice, morbid imagination, and the desire for notoriety. The anti-vivisectionists have given to the world overwhelming proof that they have not the slightest conception of the work of experimental science. Their statements are conclusive evidence that they even fail to distinguish between physiology and anatomy. But although the anti-vivisectionists continually parade their ignorance in print, they at the same time impudently proclaim themselves as final authorities in experimental science. Their methods of collecting sensational material would shame a newspaper fakir. The literature of the anti-vivisectionists is insanely morbid. Judging from the subject-matter and style, this literature seems to have two motives. The chief motive is the desire, by means of sensational misrepresentations, to incite the uneducated classes into antagonism against modern science and legitimate medicine, and incidentally to exalt fanaticism and quackery. The second motive is to suggest certain unheard-of crimes to the ill-trained youth, in order that, when these suggestions have been carried out, they may relate these crimes as the results of the scientific method of investigators. Such methods on the part of the anti-vivisectionists have called forth the following remarks in *Nature*:[1]

"The chief source of regret to us is that organizations like the Anti-vivisection Society, existing as they do upon the gullibility of an ill-informed public, should be permitted to publish their irresponsible accusations without fear of punishment. It would be better for humanity as well as science if such societies were not allowed to exist."

